# New data on trophic associations of dung beetles (Coleoptera, Scarabaeidae, Scarabaeinae) and lemurs (Primates, Lemuroidea) in Madagascar revealed by metabarcoding

**DOI:** 10.3897/BDJ.12.e130400

**Published:** 2024-08-15

**Authors:** Andrey V. Frolov, Maria S. Vishnevskaya, Heidi Viljanen, Olivier Montreuil, Lilia A. Akhmetova

**Affiliations:** 1 Zoological Institute RAS, Saint Petersburg, Russia Zoological Institute RAS Saint Petersburg Russia; 2 Saint Petersburg State University, Saint Petersburg, Russia Saint Petersburg State University Saint Petersburg Russia; 3 Finnish Museum of Natural History, Helsinki, Finland Finnish Museum of Natural History Helsinki Finland; 4 Département AVIV, UMR 7179, MECADEV, MNHN/CNRS, Entomologie, Paris, France Département AVIV, UMR 7179, MECADEV, MNHN/CNRS, Entomologie Paris France

**Keywords:** amplicon sequencing, next-generation sequencing, metabarcoding, coprophagy, food chains, gut content analysis, scarabaeines

## Abstract

Resource use and diet specialisation of Madagascan dung beetles have been little studied especially concerning the possible associations between specific dung beetle and lemur species. Pilot studies have demonstrated that amplicon sequencing is a promising tool for the lemur inventories. In the present contribution, we report the results of the gut content analysis of three endemic Madagascan dung beetles species: *Helictopleurusclouei* (Harold), *Epilissusapotolamproides* (Lebis) and *Nanosdubitatus* (Lebis). Amplicon metagenomics revealed trophic associations of these species with *Eulemursanfordi* (Archbold), *Eu.fulvus* (É.Geoffroy Saint-Hilaire) and *Cheirogaleuscrossleyi* (Grandidier), respectively. The reads of other mammal species, revealed by the analysis, including putative contaminations, are discussed.

## Introduction

Madagascar has long been recognised for its exceptional biodiversity and high level of endemism, yet it has experienced severe primary vegetation loss due to anthropogenic pressures. Since the concept of biodiversity hotspots was introduced, Madagascar was ranked first in terms of the five most important factors, including endemic taxa per area ratio and percentage of remaining primary vegetation (Myers et al. 2000). Dung beetles (Scarabaeidae, Scarabaeinae) are important elements in the food webs of ecosystems in Madagascar, where they originally evolved as consumers of lemur excrements (Wirta and Montreuil 2008, Wirta et al. 2008, Viljanen et al. 2010b, Wirta et al. 2010). The increased loss of primary forests, where the majority of dung beetles live, as well as introduction of non-native mammals, may result in significantly re-arranged tropical food chains (Hanski et al. 2008, Rahagalala et al. 2009, Wirta et al. 2014).

The study of the dung beetle trophic associations and feeding behaviour was so far mostly based on field surveys with standardised trapping protocols (Raine and Slade 2019). The development of the new generation sequencing (NGS) provides a new possibility to examine the diet of beetles by sequencing multiple DNA copies extracted from their gut content. A few pilot studies demonstrated the usage of amplicon metagenomics to investigate the food sources for dung beetles (Gómez and Kolokotronis 2017, Kerley et al. 2018, Drinkwater et al. 2021, Frolov et al. 2023, Nimalrathna et al. 2023). The amplicon metagenomic method was shown to be a promising tool for the lemur inventories in Madagascar (Frolov et al. 2023). It will also allow evaluating dung beetle capacity to shift towards other food resources and, thus, providing a better understanding of the consequences of the biodiversity crisis in Madagascar. However, the trophic associations of different dung beetle species and their food producers are still poorly known. The present paper is a contribution to our better understanding of the trophic associations of native Madagascan dung beetles, based on amplicon metagenomic analysis of the gut content of three species.

## Material and methods

### Sampling localities, material and collecting methods

**Figure 1.**
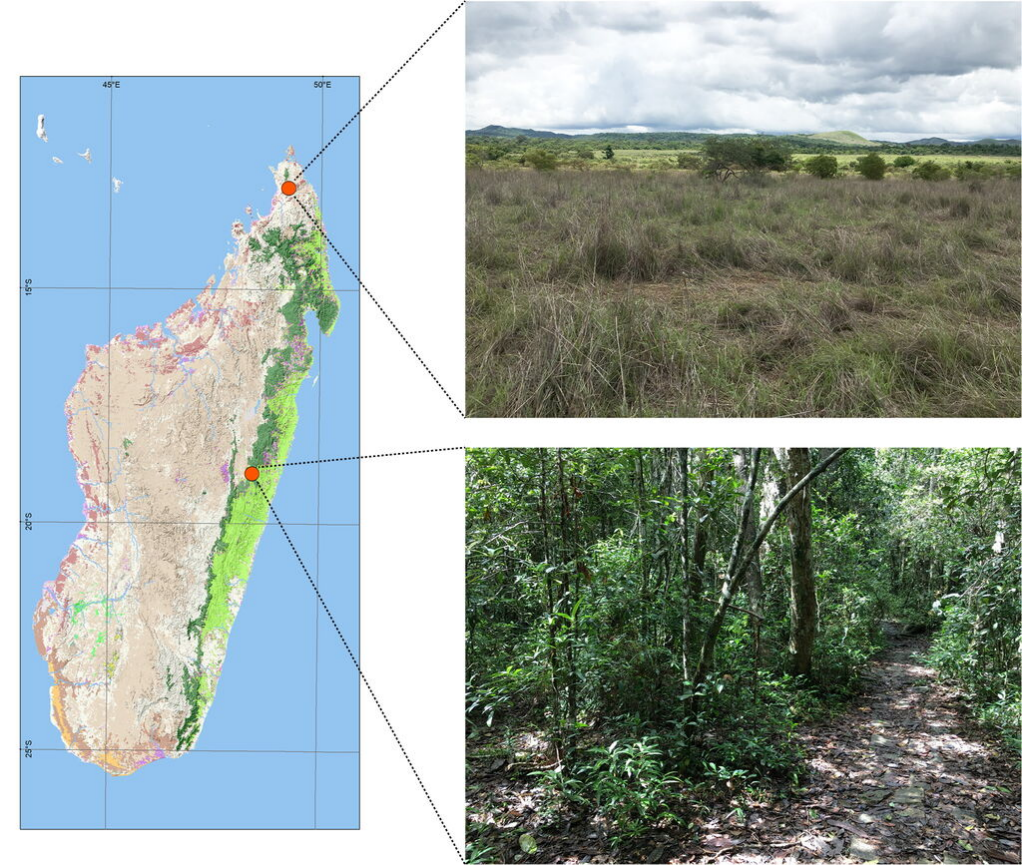
Madagascar, locality map (left) and habitats (right). Upper - Antsiranana, Ankarana Special Reserve, open area near Manongarivo; bottom - Toamasina, Analamazaotra Special Reserve.

Beetles were collected in two localities in central and northern Madagascar (Fig. 1) under a permit no 443/21/MEDD/SG/DGGE/DAPRNE/SCBE.Re. The localities are described in detail by Akhmetova et al. (2023). Voucher specimens are housed in the collection of the Zoological Institute, Saint Petersburg, Russia (ZIN) and the National Museum of Natural History, Paris, France (MNHN).

*Helictopleurusclouei* (Harold 1869)

Madagascar • Antsiranana: Ankarana Special Reserve, open area near Manongarivo, 12°51'55"S 49°13'24"E, cow dung, 6.II.2022, A.V.Frolov leg., one female (ZIN).

*Epilissusapotolamproides* (Lebis, 1961)

Madagascar • Toamasina: Analamazaotra Special Reserve, primary forest, 18°55'59"S 48°25'12"E, pitfall traps baited with human faeces, 17-20.II.2022, A.V.Frolov leg., one female (ZIN).

*Nanosdubitatus* (Lebis, 1953)

Madagascar • Toamasina: Analamazaotra Special Reserve, primary forest, 18°55'59"S 48°25'12"E, pitfall traps baited with human faeces, 17-20.II.2022, A.V.Frolov leg., one male (MNHN), two females (ZIN).

The beetles were collected from cow dung and by pitfall traps baited with human faeces. A pitfall trap was a 1-litre plastic container 10 cm in diameter buried in soil. A bait was placed in a 5 cm diameter cup wrapped in gauze and suspended by a wire above a collecting container. To avoid flooding, the traps were covered with plastic lids attached by wooden sticks about 4 cm above the ground. Funnels were placed over the collecting jars, so the beetles attracted to the traps fell into the jars and stayed alive until retrieval. The traps were exposed overnight. After retrieval, the beetles were placed in containers with 96% ethanol and transported to the laboratory after two or three weeks at room temperature; the alcohol was changed twice. The hind guts were dissected under a stereomicroscope and placed in Eppendorf tubes with 96% ethanol. To minimise possible contamination during DNA extraction stage, we used sterile disposable plastics (pipette tips with filters, Eppendorf tubes) and syringe needles. Non-disposable laboratory equipment was sterilised by hydrochloric acid solution. Before dissecting, the beetles with opened elytra were thoroughly rinsed in distilled water. Attempts were made to extract mostly guts without surrounding tissues.

### DNA extraction and sequencing

DNA was extracted from the beetle hind guts using phenol-chloroform method according to the standard protocol (Sambrook and Russell 2006). The guts with their content were homogenised in lysis buffer [25 mM EDTA, 75 mM NaCl, 10 mM Tris (pH 7.5)]. Then proteinase K (20 mg/ml) and 10% SDS were added and the samples were incubated for 2 h at 60°C. DNA was extracted from lysate first with phenol/chloroform (1:1) and then with chloroform to remove any remaining phenol. DNA was precipitated with isopropyl alcohol in the presence of 0.1 M NaCl and pelleted by centrifugation. The pellets were washed with 70% ethanol, dried and dissolved in ddH2O. The extracted DNA was stored at -20°C. The extracted DNA was quantified using a Qubit fluorimeter 4.0 with high-sensitivity reagents (Lumiprobe QuDye dsDNA HS Assay Kit) and 1 µl of DNA. Three samples with the highest DNA concentration were used for high throughput sequencing along with a control sample (distilled water). For amplicon metagenomic sequencing, the following primer pair was used: 16Smam1 (5’-CGGTTGGGGTGACCTCGGA-3’) and 16Smam2 (5’-GCTGTTATCCCTAGGGTAACT-3’). These primers amplify a short (90–95 bp) yet informative region of lrRNA and were designed to be specific for mammals (Taylor 1996). They were successfully used in a few recent works (Drinkwater et al. 2021, Ji et al. 2022, Frolov et al. 2023). NGS libraries were prepared using the NEBNext Ultra II DNA Library Prep Kit, checked with Qubit (high-sensitive reagents) and real-time PCR for quantification and Bioanalyzer for size distribution detection. The amplicon paired-end libraries (PE250) targeting an insert size of 350 bp were sequenced on Illumina NovaSeq 6000 platform aiming for 30K raw tags per sample. DNA extraction was performed at Chromas Core Facility, Saint Petersburg State University (Peterhoff, Russia), Research Park and library preparation, quality control and sequencing were performed at Novogene (Cambridge, UK). The data presented in the study are deposited in the NCBI Sequence Read Archive (SRA) database, accession numbers SRR29179174–SRR29179176.

### Bioinformatics methods

Demultiplexed raw paired reads were merged and quality filtered with usearch v.11 software (Edgar and Flyvbjerg 2015). Primers were trimmed and the reads were quality filtered with the -fastq_maxee 1.0 option. The reads were re-labelled to add sample identifiers and pooled to enhance sensitivity of analysis. OTU (operational taxonomic unit) analysis was carried out with two approaches implemented by usearch v.11: UPARSE (generating OTUs by clustering reads with 97% similarity) (Edgar 2013) and UNOISE (generating ZOTUs, based on error-correction) (Edgar and Flyvbjerg 2015) with the -minsize 11 option. Both methods produced similar results, with the total number of OTUs being slightly smaller than ZOTUs; therefore, OTUs were used for downstream analysis. OTUs were then manually annotated with BLAST (https://blast.ncbi.nlm.nih.gov) using megablast algorithm against nucleotide database.

## Results

Illumina sequencing yielded 247816 reads for three samples; negative control yielded no reads. After merging and quality filtering, we obtained 205762 clean reads (over 83% of raw reads). Analysis with usearch revealed 48 OTUs and 52 ZOTUs. Statistics per sample are given in Table 1.

After annotation of OTUs with BLAST, 14 of them returned no hits, 17 ZOTUs were annotated as human pseudogenes (numts) and five OTUs were attributed to non-mammalian species. After these OTUs were discarded, 12 OTUs were retained which comprised 193069 reads. They belonged to eleven mammal species (Table 2).

## Discussion

Resource use or diet specialisation of Madagascan dung beetles has been little studied especially concerning the possible associations between specific dung beetle and lemur species. Viljanen (2004) conducted the first study on the topic in Ranomafana National Park by using lemur faeces baited pitfall traps to attract dung beetles. The Park is located within the eastern wet forest belt in SE Madagascar. The study included faeces of seven lemur species that occur in the Park (omnivorous *Microcebusrufus* (Geoffroy), frugivorous *Eulemurrubriventer* (Geoffroy), vegetarian large bodied *Propithecusedwardsi* Grandidier and three bamboo-eating species that belong to the genus *Hapalemur* Geoffroy at that time). In addition to lemur faeces-baited traps, fish, meat of pig and zebu-cattle, rotten fruit and cattle dung-baited traps were used. However, no clear associations of specific dung beetle and lemur species were found. To date, the only known six individuals of *Epilissusgenieri* Montreuil were trapped solely with the *Hapalemuraureus* (Meier) faeces-bated traps. However, due to trapping design, this result maybe an artefact. In addition, small-bodied *Helictopleurussemivirens* Lebis seemed to prefer the faeces of the largest-bodied lemur species *P.edwardsi*. In Madagascar, the general pattern seems to be that half of wet forest inhabiting endemic dung beetle species are either carrion feeders or generalist feeding on both carrion and faeces and the other half might be considered dung specialists excluding cattle dung (Viljanen et al. 2010b). Dung specialist species seem to prefer faeces of different lemur species in relation to their size and diet – large-bodied vegetarian or frugivorous species were preferred over bamboo-eating or omnivorous species (Viljanen 2004). However, most of the dung specialist species are easily collected with human faeces-baited traps. Human faeces most likely act as a substitute for the faeces of extinct large-bodied lemur species. Indeed, altogether 46 dung beetle species of the known 285 species have been trapped with human faeces-bated traps in Madagascar (Viljanen, unpublished data). The totally new type of resource, cattle dung, is not used by wet forest dwelling dung beetle species, but interestingly, several endemic western dry forest species have switched their habitat and resource use from forest and carrion or lemur faeces to open areas and cattle dung within the last 1,500 years. These species have been able spread across the island (Hanski et al. 2008, Rahagalala et al. 2009). In comparison, wet forest species have much more limited ranges along the eastern wet forest belt, the species turnover (beta-diversity) being extremely high compared to other tropical areas (Viljanen et al. 2010a).

In the pilot study using amplicon metagenomic analysis of Madagascan dung beetles, Frolov et al. (2023) provided evidence of trophic association of four dung beetle species and six lemur species. The present results are congruent with the previous work. The majority of reads from two samples of the three belonged to human and cow. Both food sources are available in the sampling areas. The exception is *H.clouei* specimen, where over 40% of total reads belonged to small Indian civet (*Viverriculaindica* Geoffroy). The civet DNA was also found in *E.apotolamproides* sample, although in a smaller number. Small Indian civet is non-native to Madagascar, but is now widely distributed on the island (Shehzad et al. 2012).

The reads of house mouse (*Musmusculus* Linnaeus) and pig (*Susscrofa* Linnaeus) may be a result of contamination or indicate a real, though probably occasional, feeding on the faeces of these animals. It was shown that the DNA of domestic and synanthropic animals could be present in PCR reagents (Leonard et al. 2007). On the other hand, these non-native animals are now widely distributed in Madagascar. The *N.dubitatus* sample yielded reads of Norway lemming (*Lemmuslemmus* (Linnaeus)) and *H.clouei* and *E.apotolamproides* yielded reads of Bay duiker (*Cephalophusdorsalis* Gray). Both species do not occur in Madagascar and their reads apparently resulted from contamination. Cat (*Feliscatus* Linnaeus) is now widespread in Madagascar, but feeding on faeces of carnivorous animals was not registered and considering the small number of cat reads, we think that they may have resulted from contamination.

Two lemur species were revealed by the annotation of the OTUs with GenBank database: brown lemur (*Eulemurfulvus* (Geoffroy)) and furry-eared dwarf lemur (*Cheirogaleuscrossleyi* Grandidier). First, species DNA was found in the gut content of *E.apotolamproides* and *H.clouei* and second – in *N.dubitatus*. Brown lemur was considered a complex of 6–7 subspecies which were elevated to full species by Groves (2006). Brown lemur has a disjunct range with the northern part being a small area south of Beramanja (Mittermeier et al. 2008), which is some 70 km south-west of the collecting locality of *H.clouei*. Brown lemur is replaced with Sanford's lemur in the northernmost Madagascar. Wilson et al. (1988) wrote that Sanford's lemur (treated a subspecies of brown lemur) was present in substantial numbers at Ankarana and favoured degraded forest or forest bordering the savannah surrounding the massif. This agrees well with the biotope where *H.clouei* was collected. The data available in GenBank (checked 27.11.2023) do not allow differentiating the two species, based on the amplified fragment, probably due to the close relationships of the taxa. However, considering the well-studied distribution of lemurs, we consider that the reads from *H.clouei* belong to Sanford's lemur and those from *E.apotolamproides* belong to brown lemur. Furry-eared dwarf lemur is distributed in central-east Madagascar, mostly in the rainforest belt from Andasibe-Mantadia National Park in the south to Zahamena National Park in the north (Lei et al. 2014). Therefore, the collecting localities of sample specimens agree well with known distribution of lemurs.

**Figure 2.**
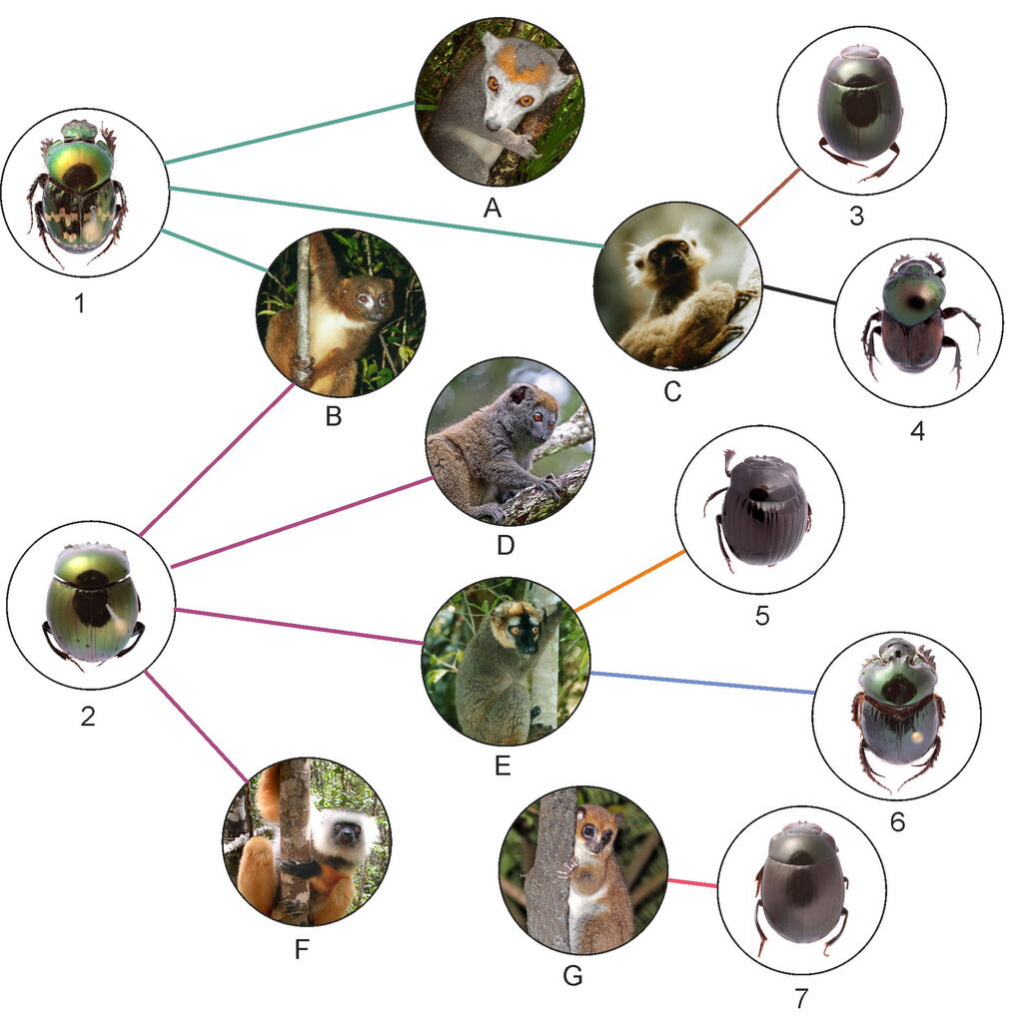
Trophic associations of scarabaeinae dung-beetles and lemurs registered through metabarcoding of the beetles gut content. 1 *Helictopleurusfissicollis* (Fairmaire); 2 *Epilissussplendidus* Fairmaire; 3 *Nanosagaboides* (Boucomont); 4 *Helictopleurusclouei* (Harold); 5 *Epilissusapotolamproides* (Lebis); 6 *Helictopleurusgiganteus* (Harold); 7 *Nanosdubitatus* (Lebis); A *Eulemurcoronatus* (Gray); B *Eulemurrubriventer* (I.Geoffroy Saint-Hilaire); C *Eulemursanfordi* Archbold; D *Hapalemurgriseus* (Link); E *Eulemurfulvus* (É.Geoffroy Saint-Hilaire); F *Propithecusdiadema* Bennett G *Cheirogaleuscrossleyi* A.Grandidier.

To date, trophic associations of seven dung beetle species with seven lemur species are registered by metabarcoding analysis of the beetles gut content (Fig. 2). These results show that a beetle species may feed on faeces of different lemur species. There seems to be little specificity in the food preference amongst dung-beetle species. However, these results are based on the limited material and apparently represent a part of a rather complex food web. Further research is needed to clarify the trophic associations of different species and the metabarcoding methods proved a suitable tool for such research.

The trophic associations of Madagascan dung beetles and wild mammals registered by metabarcoding are also reported as nanopublications associated with this work. Nanopublications are small data containers represented by named RDF graphs that can be automatically interpreted and aggregated (Kuhn et al. 2016) and can be used in biodiversity research for communicating new information in a standardised way (Dimitrova et al. 2021). We think that such nanopublications may be especially useful in accumulating and analysing data in food webs studies.

## Figures and Tables

**Table:** 

Nanopublication	Creator	Date
https://w3id.org/kpxl/pensoft/bdj/np/RAeZiKuzUVaj2OF7DAldEmLrVeWGAi_ubFQO2MEj7TQu8	Andrey Frolov	15-08-2024 07:15:43
https://w3id.org/kpxl/pensoft/bdj/np/RAHOQYQpJKZploIdfqN_eA3jVCmBytEl9Bv3wbf808ER8	Andrey Frolov	15-08-2024 07:16:29
https://w3id.org/kpxl/pensoft/bdj/np/RA_tmhVXWkT4ri-4ioTaNXtjj9Z2KuO63URlgydM6jM0g	Andrey Frolov	15-08-2024 07:17:18
https://w3id.org/kpxl/pensoft/bdj/np/RAniX6TOU3YqOXep93ftX_Abiz-U7clo0Z0em0_acybrg	Andrey Frolov	15-08-2024 07:17:51
https://w3id.org/kpxl/pensoft/bdj/np/RA3ANtGf_u5XOys_OgoTJa9eelGSC8I4xoq5bk0631CR0	Andrey Frolov	15-08-2024 07:18:25
https://w3id.org/kpxl/pensoft/bdj/np/RARg-S2n0Scc7r0bCtrlSKLGEYwt2C4ocQa3dea2rwfuo	Andrey Frolov	15-08-2024 07:19:10
https://w3id.org/kpxl/pensoft/bdj/np/RABm0KuttCDIFV71Q0FAGXOJR4lzZiDYaVsFSPC7g2a5k	Andrey Frolov	15-08-2024 07:19:44
https://w3id.org/kpxl/pensoft/bdj/np/RAc8TbgA6DYqbaEGadIxxxYL3BFDsbdSeW0aUauln2vdw	Tobias Kuhn	15-08-2024 07:20:25
https://w3id.org/kpxl/pensoft/bdj/np/RApI_vjABMHc6PRlaSDxepHOIY_my0py97Rlm_tiB2Qqs	Andrey Frolov	15-08-2024 07:21:13
https://w3id.org/kpxl/pensoft/bdj/np/RA77p6DyHmtPQfo0VkeVi-RNzvKOfgxPoP00B-N5gUgxY	Andrey Frolov	15-08-2024 07:25:56
https://w3id.org/kpxl/pensoft/bdj/np/RAJlgtundyB7Xnu8KnRm-TxZfUA1mMP2lDi0YzidiuJ1E	Andrey Frolov	15-08-2024 07:25:19
https://w3id.org/kpxl/pensoft/bdj/np/RA5Im6r1SafK5_DjiL1GvZDJ32Ed1w6k7oUt_A6Oane50	Andrey Frolov	15-08-2024 07:24:31
https://w3id.org/kpxl/pensoft/bdj/np/RAJ7KNmBW8fgbzGooDKxQLPPaH-F9I84khE7vox-FzylM	Andrey Frolov	15-08-2024 07:23:57
https://w3id.org/kpxl/pensoft/bdj/np/RAt_S1PP8it28_B5ht-RbSAZr8dZrj5-uHKOzWrVH2H38	Andrey Frolov	15-08-2024 08:40:46

**Table 1.** Statistics and bioinformatics results of amplicon metagenomics analysis of gut content of the three specimens of *H.clouei*, *E.apotolamproides* and *N.dubitatus*.

**Parameter \ Sample**	***H.clouei***	***E.apotolamproides***	***N.dubitatus***
raw reads	92824	89540	65452
merged reads	75383	88362	42050
clean reads	75373	88345	42044
OTUs	37	32	21
ZOTUs	38	33	20

**Table 2.** Results of OTUs annotation with BLAST (see Discussion regarding *Eu.sanfordi*). Data are given as a number (#) and percent (%) of reads per sample.

Mammal species	Sample (beetle specimen)							
	*H.clouei*		*E.apotolamproides*				*N.dubitatus*	
	#	%		#	%	#		%
*Homosapiens*	13362	19		60421	74	11352		29
*Bostaurus*	18495	26		17766	22	24718		62
*Viverriculaindica*	30767	43		695	1	0		0
*Musmusculus*	8093	11		163	0	0		0
*Eulemurfulvus*	0	0		2752	3	0		0
*Eulemursanfordi*	60	0		0	0	0		0
*Lemmuslemmus*	0	0		0	0	1897		5
*Cheirogaleuscrossleyi*	0	0		0	0	1059		3
*Susscrofa*	702	1		4	0	522		1
*Cephalophusdorsalis*	5	0		182	0	0		0
*Feliscatus*	1	0		53	0	0		0
Total	71485	100		82036	100	39548		100

## References

[B10904806] Akhmetova Lilia A, Montreuil Olivier, Frolov Andrey V (2023). Diversity of the endemic Madagascan dung beetles (Coleoptera, Scarabaeidae, Scarabaeinae): new records from six protected areas. Diversity.

[B11744967] Dimitrova Mariya, Georgiev Teodor, Penev Lyubomir (2021). A nano(publication) approach towards big data in biodiversity. Biodiversity Information Science and Standards.

[B10904867] Drinkwater Rosie, Williamson Joseph, Clare E. L, Chung A Y C, Rossiter S J, Slade Eleanor (2021). Dung beetles as samplers of mammals in Malaysian Borneo—a test of high throughput metabarcoding of iDNA. PeerJ.

[B10904824] Edgar Rt C (2013). UPARSE: highly accurate OTU sequences from microbial amplicon reads. Nature Methods.

[B10904815] Edgar R C, Flyvbjerg Henrik (2015). Error filtering, pair assembly and error correction for next-generation sequencing reads. Bioinformatics.

[B10904833] Frolov Andrey V, Akhmetova Lilia A, Vishnevskaya Maria S, Kiriukhin Bogdan A, Montreuil Olivier, Lopes Fernando, Tarasov Sergei I (2023). Amplicon metagenomics of dung beetles (Coleoptera, Scarabaeidae, Scarabaeinae) as a proxy for lemur (Primates, Lemuroidea) studies in Madagascar. ZooKeys.

[B10904788] Gómez Andrés, Kolokotronis Sergios-Orestis (2017). Genetic identification of mammalian meal source in dung beetle gut contents. Mitochondrial Dna Part A.

[B10904749] Groves CP (2006). Red-fronted lemurs are not the same as red lemurs. Australasian Primatology.

[B10904964] Hanski Ilkka, Wirta Helena, Nyman Toshka, Rahagalala Pierre (2008). Resource shifts in Malagasy dung beetles: contrasting processes revealed by dissimilar spatial genetic patterns. Ecology Letters.

[B10904887] Ji Yinqiu, Baker C. C. M., Popescu Viorel D., Wang Jiaxin, Wu Chunying, Wang Zhengyang, Li Yuanheng, Wang Lin, Hua Chaolang, Yang Zhongxing, Yang Chunyan, Xu Charles C. Y., Diana Alex, Wen Qingzhong, Pierce Naomi E., Yu Douglas W. (2022). Measuring protected-area effectiveness using vertebrate distributions from leech iDNA. Nature Communications.

[B10904932] Kerley G. I. H., Landman Marietjie, Ficetola G. F., Boyer Frédéric, Bonin Aurélie, Rioux Delphine, Taberlet Pierre, Coissac Eric (2018). Diet shifts by adult flightless dung beetles *Circelliumbacchus*, revealed using DNA metabarcoding, reflect complex life histories. Oecologia.

[B11744943] Kuhn Tobias, Chichester Christine, Krauthammer Michael, Queralt-Rosinach Núria, Verborgh Ruben, Giannakopoulos George, Ngonga Ngomo Axel-Cyrille, Viglianti Raffaele, Dumontier Michel (2016). Decentralized provenance-aware publishing with nanopublications. PeerJ Computer Science.

[B10904773] Lei Runhua, Frasier Cynthia L, McLain Adam T, Taylor Justin M, Bailey Carolyn A, Engberg Shannon E, Ginter Azure L, Randriamampionona Richard, Groves Colin P, Mittermeier Russell A (2014). Revision of Madagascar's dwarf lemurs (Cheirogaleidae: Cheirogaleus): designation of species, candidate species status and geographic boundaries based on molecular and morphological data. Primate Conservation.

[B10904845] Leonard Jennifer A, Shanks Orin, Hofreiter Michael, Kreuz Eva, Hodges Larry, Ream Walt, Wayne Robert K, Fleischer Robert C (2007). Animal DNA in PCR reagents plagues ancient DNA research. Journal of Archaeological Science.

[B10904758] Mittermeier Russell A, Ganzhorn Jörg U, Konstant William R, Glander Kenneth, Tattersall Ian, Groves Colin P, Rylands Anthony B, Hapke Andreas, Ratsimbazafy Jonah, Mayor Mireya I (2008). Lemur diversity in Madagascar. International Journal of Primatology.

[B10904922] Myers Norman, Mittermeier Russell A, Mittermeier Cristina G, Da Fonseca Gustavo AB, Kent Jennifer (2000). Biodiversity hotspots for conservation priorities. Nature.

[B11744499] Nimalrathna Thilina S., Fan Huan, Quan Rui-Chang, Nakamura Akihiro (2023). Enhancing the dung beetle iDNA tool for mammalian biodiversity monitoring and ecological studies. Integrative Conservation.

[B10904993] Rahagalala P, Viljanen Heidi, Hottola Jenni, Hanski I (2009). Assemblages of dung beetles using cattle dung in Madagascar. African Entomology.

[B10904797] Raine Elizabeth H, Slade Eleanor M (2019). Dung beetle–mammal associations: methods, research trends and future directions. Proceedings of the Royal Society B.

[B10905002] Sambrook Joseph, Russell D W (2006). Purification of nucleic acids by extraction with phenol: chloroform. Cold Spring Harbor Protocols.

[B10904908] Shehzad Wasim, Riaz Tiayyba, Nawaz M A, Miquel Christian, Poillot Carole, Shah S A, Pompanon Francois, Coissac Eric, Taberlet Pierre (2012). Carnivore diet analysis based on next-generation sequencing: Application to the leopard cat (*Prionailurusbengalensis*) in Pakistan. Molecular Ecology.

[B10904878] Taylor P G (1996). Reproducibility of ancient DNA sequences from extinct Pleistocene fauna. Molecular Biology and Evolution.

[B10904741] Viljanen Heidi (2004). Diet specialization among endemic forest dung beetles in Madagascar. MSc Thesis.

[B10904984] Viljanen Heidi, Escobar Federico, Hanski Ilkka (2010). Low local but high beta diversity of tropical forest dung beetles in Madagascar. Global Ecology and Biogeography.

[B10905011] Viljanen Heidi, Wirta Helena, Montreuil Olivier, Rahagalala Pierre, Johnson Steig, Hanski Ilkka (2010). Structure of local communities of endemic dung beetles in Madagascar. Journal of Tropical Ecology.

[B10904858] Wilson Jane M, Stewart Paul D, Fowler Simon V (1988). Ankarana—a rediscovered nature reserve in northern Madagascar. Oryx.

[B10905022] Wirta Helena, Montreuil Olivier (2008). Evolution of the *Canthoninilongitarsi* (Scarabaeidae) in Madagascar. Zoologica Scripta.

[B10904945] Wirta Helena, Orsini Luisa, Hanski Ilkka (2008). An old adaptive radiation of forest dung beetles in Madagascar. Molecular Phylogenetics and Evolution.

[B10904954] Wirta Helena, Viljanen Heidi, Orsini Luisa, Montreuil Olivier, Hanski Ilkka (2010). Three parallel radiations of Canthonini dung beetles in Madagascar. Molecular Phylogenetics and Evolution.

[B10904973] Wirta H. K., Hebert P. D.N., Kaartinen Riikka, Prosser S. W., Várkonyi Gergely, Roslin Tomas (2014). Complementary molecular information changes our perception of food web structure. Proceedings of the National Academy of Sciences.

